# Perspectives on the implications of carrying putative pathogenic variants in the medulloblastoma predisposition genes *ELP1* and *GPR161*

**DOI:** 10.1007/s10689-023-00330-7

**Published:** 2023-03-24

**Authors:** Miriam J Smith, Emma R Woodward, D Gareth Evans

**Affiliations:** 1 Division of Evolution, Infection and Genomics, School of Biological Sciences, Faculty of Biology, Medicine and Health, University of Manchester, Manchester, UK; 2 Manchester Centre for Genomic Medicine, Manchester Academic Health Science Centre, St Mary’s Hospital, Manchester University NHS Foundation Trust, M13 9WL Manchester, UK

**Keywords:** *ELP1*, *GPR161*, Medulloblastoma, Gorlin Syndrome

## Abstract

Recent genetic sequencing studies in large series’ of predominantly childhood medulloblastoma have implicated loss-of-function, predominantly truncating, variants in the *ELP1* and *GPR161* genes in causation of the MB_SHH_ subtype specifically. The latter association, along with a report of an index case with some features of Gorlin syndrome has led to speculation that *GPR161* may also cause Gorlin syndrome. We show that these genes are associated with relatively low absolute risks of medulloblastoma from extrapolating lifetime risks in the general population and odds ratios from the population database gnomAD. The projected risks are around 1 in 270–430 for *ELP1* and 1 in 1600–2500 for *GPR161.* These risks do not suggest the need for MRI screening in infants with *ELP1* or *GPR161* variants as this is not currently recommended for *PTCH1* where the risks are equivalent or higher. We also screened 27 *PTCH1/SUFU* pathogenic variant-negative patients with Gorlin syndrome for *GPR161* and found no suspicious variants. Given the population frequencies of 0.0962% for *GPR161* and 0.0687% for *ELP1*, neither of these genes can be a cause of Gorlin syndrome with an unexplained population frequency far lower at 0.0021%.

**Supplementary Information:**

The online version contains supplementary material available at 10.1007/s10689-023-00330-7.

## Short report

Medulloblastoma is a relatively rare malignant brain tumour that has a main peak of incidence in early childhood and infancy [1, 2]. The main syndromic predisposition to medulloblastoma is Gorlin syndrome which is characterised by multiple skin basal cell carcinoma, macrocephaly and jaw cysts with two proven gene associations (*PTCH1* and *SUFU*) accounting for ~ 70% of cases [3]. Recent genetic sequencing studies in large series of predominantly childhood medulloblastoma have implicated loss-of-function, predominantly truncating, variants in the *ELP1* (mean age of onset = 6.5years accounts for 3.2% of cases) and *GPR161* (mean age of onset = 1.5years; accounts for 0.38% of cases) genes[4,5-Table 1] in causation of the MB_SHH_ subtype specifically. The latter association, along with a report of an index case with some features of Gorlin syndrome (basal cell carcinomas, frontal bossing and a meningioma, but microcephaly instead of macrocephaly), has led to speculation that *GPR161* may also cause Gorlin syndrome [5].

As reported in both papers [4, 5], the frequency of presumed loss-of-function variants in the population database gnomAD (https://gnomad.broadinstitute.org/) is high for a rare condition with both reports showing incidence above the rare disease threshold of 1 in 2,000-(Table 1). Neither paper attempted to assess the overall likelihood of a child with either an *ELP1* or *GPR161* loss-of-function variants developing medulloblastoma. In view of the likelihood that these genes will be added to multi-gene panels for childhood malignancy and possibly for Gorlin syndrome assessment, we have assessed the overall likelihood of medulloblastoma with each gene and specifically tested for *GPR161* variants in our currently unexplained Gorlin syndrome families.

**Table 1:** Cases of medulloblastoma annually in USA and UK and implied overall childhood risk with inferred odds ratios and childhood risk with *ELP1* and *GPR161* variants

	UK	USA
medulloblastoma cases annually in children	55 [1]	500 [2]
Children in UK/USA [ref 1,2 below table]	12,700,000	72,822,113
16-year number of medulloblastoma	880	8000
% of children who develop MB	0.007%	0.011%
frequency	1 in 14431.8	1 in 9102.8
Proportion of MB associated with germline *ELP1 PV* [4]	23/713 (3.23%)	23/713 (3.23%)
Controls in gnomAD with ELP1 [from ref 4]	114	114
All tested in gnomAD [from ref 4]	118,479	118,479
% with ELP1 in general population	0.0962%	0.0962%
Odds Ratio of MB in ELP1	33.53	33.53
Chance of developing childhood MB with *ELP1*	0.23%	0.37%
1 in x chance of developing childhood (< 16y) MB with *ELP1*	1 in 430.4	1 in 271.5
Proportion of MB associated with germline *GPR161* PV	4*/1040 (0.38%	4*/1040 (0.38%)
Controls in gnomAD with ELP1	86	86
All tested in gnomAD	125,153	125,153
% with *GPR161*	0.069%	0.069%
OR of MB in *GPR161*	5.60	5.60
Chance of developing childhood MB with *GPR161*	0.04%	0.06%
1 in x chance of developing childhood (< 16y) MB with *GPR161*	1 in 2578.4	1 in 1626.3

*Excludes missense variant as only compared to truncating variants. Also excludes index case as not clear from series of 1040 cases. PV-pathogenic variant, MB-medulloblastoma; OR Odds Ratio

1. https://www.ons.gov.uk/peoplepopulationandcommunity/populationandmigration/populationestimates/bulletins/annualmidyearpopulationestimates/mid2019estimates

2. https://www.ojjdp.gov/ojstatbb/population/qa01104.asp

## Methods

The population likelihood of medulloblastoma was assessed using data sources on annual rates of childhood medulloblastoma [1, 2] and number of children in the UK and USA. Relative risk of medulloblastoma was assessed from presumed loss-of-function variants for each gene in the published medulloblastoma series divided by the frequency in gnomAD as previously described [3].

We also sequenced the *GPR161* gene (NM_001375883.1) in 27 people with Gorlin syndrome who had previously been screened for variants in *PTCH1* and *SUFU* and in whom no causative variant had been found.

## Results

Table 1 shows the results of our analysis on likelihood of childhood medulloblastoma assuming 16 years of risk in childhood. The likelihood varied from 1 to 9,000 in the USA to 1 in 14,000 in the UK. We took the derived gnomAD population data from the *ELP1* paper for loss-of-function variants showing a very high population frequency of close to 1 in 1000-(Table). Thus, despite a 3.2% frequency of *ELP1* loss-of-function variants in medulloblastoma, this only resulted in a relative risk of 33.5 and a childhood risk of 1/ 270-1/430 for medulloblastoma-(Table 1-row 13). For *GPR161* we found 86 loss-of-function variants, including canonical splicing variants, in an average of 125,153 individuals in gnomAD. We excluded the putative missense variant in *GPR161* and the index case leaving only 4 definite loss-of-function variants amongst 1040 cases. This resulted in a relative risk of only 5.6-fold and childhood risk of 1 in 1600–2500 in the USA/UK. We did not assess the likelihood for the MB_SHH_ subtype separately. However, as all but one of the cases was in the MB_SHH_ subtype and the frequency in non SHH pathway medulloblastoma for *ELP1* was only 1/542 (0.18%), similar to the 0.1% frequency in controls, we have assumed that all of the risk related to the MB_SHH_ subtype.

Our specific screen of *GPR161* in our cohort of 27 *PTCH1*/*SUFU* pathogenic variant-negative patients with Gorlin syndrome found no pathogenic or likely pathogenic variants.

## Discussion

It is likely parents of children are already receiving results indicating that their child has loss-of-function variant in *ELP1* or *GPR161*. Indeed, in England neonates can now undergo genome sequencing at birth without any obvious symptoms and parents could receive results for an incidental pathogenic variant in *ELP1* or *GPR161* as genes associated with childhood malignancy. The present study will provide counsellors with sufficient risk information to provide an accurate risk assessment and recommendations. For instance, parents of neonates with a *GPR161* variant can be reassured that absolute risks of medulloblastoma are very small and the increased risk may disappear after age 4 years (this will be reassuring if results are given for a 5year old) similar to *SUFU* and *PTCH1* [3, 5, 6]. Whereas, parents of neonates with an *ELP1* loss-of-function variant can be told the risks are higher but not sufficient for MRI screening [3, 6] and that the increased risk lasts well beyond 7years of age. The risks for siblings of medulloblastoma cases who are heterozygotes may still justify closer monitoring especially for *ELP1* as the family may carry additional genetic factors that predispose to medulloblastoma. Without this granular information on the true implications of this finding these parents may become distressed and concerned about the requirements for clinical screening. The implied likelihood of developing medulloblastoma for both *ELP1* and particularly *GPR161* are overall quite low and at or well below that of *PTCH1* for which no screening in childhood for medulloblastoma is recommended [3, 6]. The suggestive features for a possible diagnosis of Gorlin syndrome in the index *GPR161* patient was not backed up by information on the father, brother and two nephews who were also heterozygous [2]. The population frequency of both *ELP1* and *GPR161* of close to 1 in 1,000 are also far too high to account for the small amount of missing heritability in a condition with a birth incidence of only 1 in 14,500 [7, 8]. We did not identify *GPR161* in 27 *PTCH1/SUFU* negative Gorlin syndrome families which represent all the 27/86-(31.4%) unexplained by known genes (59 were due to *PTCH1/SUFU*) meaning an unexplained population frequency far lower at 0.0021% (1 in 46,400). Given the absence of clinical data and the even higher population frequency of *ELP1* we have not assessed this gene in our Gorlin population. *ELP1* and *GPR161* therefore join *PTCH2* as potential candidate genes for Gorlin syndrome that can be dismissed on their population frequencies and, for *PTCH2* and *GPR161*, their absence in Gorlin syndrome kindreds [8].

We do not have an explanation for the apparent higher incidence of childhood medulloblastoma in the USA compared to the UK and this seems unlikely to be linked to an increased frequency of the known predisposition genes.

In examining gnomAD, we found a ClinVar entry for a putative likely pathogenic *GPR161* missense variant, c.56T > A,p.Leu19Gln;NM_001267609;chr1:g.168,074,093 A > T[hg19] (this variant is annotated as c.-5T > A; 5’-UTR in the MANE select transcript:NM_001375883.1). The variant had been reported in homozygous form in a Turkish family as causing Pituitary Stalk Interruption Syndrome [9]. Although this quite clear syndromic diagnosis tracked with zygosity in a consanguineous kindred (the unaffected heterozygote parents had 4 further unaffected children who were not homozygous) no population frequency for this variant was supplied. We have now assessed this in gnomAD and found that it is present 18 times in South Asians in homozygous form in 15,296 individuals (1 in 850 individuals) with a further 641 being heterozygote (1 in 24). This clearly cannot be a loss-of-function variant that could cause a medulloblastoma risk (particularly as homozygous) or this would mean a much higher likelihood of medulloblastoma in South Asia and the homozygote frequency is far too high to be associated with such a rare (estimated 0.5 per million) complex condition as Pituitary Stalk Interruption Syndrome [10].

In conclusion, the absolute risk of developing childhood medulloblastoma with *ELP1* and *GPR161* appears low and families can be reassured particularly if these genes are found incidentally on panels. There is still a need to identify and follow patients with medulloblastoma in order to better analyse the clinical features, outcome and risk of second malignancies associated with these variants Radiological screening for medulloblastoma appears not to be justified in incidentally identified cases but the risks for siblings of medulloblastoma cases who are heterozygotes may still justify closer monitoring. *ELP1* and *GPR161* can be dismissed as candidates for Gorlin syndrome and it now also seems unlikely that *GPR161* homozygotes have Pituitary Stalk Interruption Syndrome.

## Electronic supplementary material

Below is the link to the electronic supplementary material.

Supplementary Material 1

## Data Availability

The data that support the findings of this study are available on request from the corresponding author. The data are not publicly available due to privacy or ethical restrictions.
